# Caregiver Evaluation of the Quality of End-Of-Life Care (CEQUEL) Scale: The Caregiver's Perception of Patient Care Near Death

**DOI:** 10.1371/journal.pone.0066066

**Published:** 2013-06-06

**Authors:** Philip C. Higgins, Holly G. Prigerson

**Affiliations:** 1 Doctoral candidate, Boston College Graduate School of Social Work, Chestnut Hill, Massachusetts, United States of America; 2 Department of Psychosocial Oncology and Palliative Care, Dana-Farber Cancer Institute, Boston, Massachusetts, United States of America; 3 Center for Psychosocial Epidemiology and Outcomes Research, Dana-Farber Cancer Institute, Boston, Massachusetts, United States of America; 4 Division of Population Sciences, Department of Medical Oncology, Dana-Farber Cancer Institute, Boston, Massachusetts, United States of America; 5 Department of Psychiatry, Brigham and Women's Hospital, Boston, Massachusetts, United States of America; 6 Harvard Medical School, Boston, Massachusetts, United States of America; The University of Hong Kong, Hong Kong

## Abstract

**Purpose:**

End-of-life (EOL) measures are limited in capturing caregiver assessment of the quality of EOL care. Because none include caregiver perception of patient suffering or prolongation of death, we sought to develop and validate the Caregiver Evaluation of Quality of End-of-Life Care (CEQUEL) scale to include these dimensions of caregiver-perceived quality of EOL care.

**Patients and Methods:**

Data were derived from Coping with Cancer (CwC), a multisite, prospective, longitudinal study of advanced cancer patients and their caregivers (N = 275 dyads). Caregivers were assessed before and after patient deaths. CEQUEL's factor structure was examined; reliability was evaluated using Cronbach's α, and convergent validity by the strength of associations between CEQUEL scores and key EOL outcomes.

**Results:**

Factor analysis revealed four distinct factors: Prolongation of Death, Perceived Suffering, Shared Decision-Making, and Preparation for the Death. Each item loaded strongly on only a single factor. The 13-item CEQUEL and its subscales showed moderate to acceptable Cronbach's α (range: 0.52–0.78). 53% of caregivers reported patients suffering more than expected. Higher CEQUEL scores were positively associated with therapeutic alliance (ρ = .13; p≤.05) and hospice enrollment (z = −2.09; p≤.05), and negatively associated with bereaved caregiver regret (ρ = −.36, p≤.001) and a diagnosis of Posttraumatic Stress Disorder (z = −2.06; p≤.05).

**Conclusion:**

CEQUEL is a brief, valid measure of quality of EOL care from the caregiver's perspective. It is the first scale to include perceived suffering and prolongation of death. If validated in future work, it may prove a useful quality indicator for the delivery of EOL care and a risk indicator for poor bereavement adjustment.

## Introduction

Cancer caregivers are key stakeholders not only in active cancer care, but also in terminal care and bereavement. Caregivers provide an important perspective on, and reliable assessment of, the quality of end-of-life (EOL) care patients receive [1]. Caregiver ratings of the quality of EOL care may also have consequences for their mental health, proving a risk factor for poor bereavement adjustment.

Many have studied what it means to have a ‘good death’ [2], the distinctions between quality of life (QOL) and quality of death [3], and how best to measure the quality of care received at the EOL [4]–[7]. Research has identified factors important to dying patients and their caregivers, including avoidance of prolonged death or suffering, shared decision-making, communication with providers about patient wishes, awareness of prognosis and preparation for death [8]–[15]. Instruments designed to measure the quality of EOL care [16] usually elicit patient experiences via patient or proxy response, rather than the caregiver's experience [11], [17]–[20]. How caregivers perceive a dying loved one's care should be of concern to healthcare providers, as it is an indicator of the quality of care the team has provided and also affects caregiver bereavement [21]–[33]. Existing caregiver measures typically assess caregiver burden or QOL, but not perceived quality of care to the dying patient [34].

A key exception is the Toolkit After-Death Bereaved Family Member Interview, which measures multiple domains of caregiver-perceived quality of care in the final week of life [35]. Although the Toolkit is a broad and clinically relevant instrument that offers the best current means by which to measure caregiver evaluation of EOL care, it omits two factors identified as important to dying patients and their caregivers: perceived patient suffering and prolongation of death [8]–[15]. The Toolkit assesses perceived adequacy of symptom management, but not the meaning caregivers derive from inadequate palliation. This perceived suffering or violent harm to the patient may greatly influence caregivers' bereavement adjustment. Similarly, the Toolkit does not capture the caregiver's experience of ‘emotional limbo’ during the seemingly indefinite period of waiting for death to come. Caregivers often feel that better EOL care could have curtailed this waiting period. Bereaved caregivers report wishing that they had been better prepared for the dying process – including how long it might take – by the care team [30], [31], [36]. As our understanding of death and dying grows, there is heightened recognition of the multiple dimensions involved in caregiver evaluation of the quality of EOL care. We consider perceived suffering and prolongation of death to be two such important dimensions, the inclusion of which extends the important work of the Toolkit's authors to create a more comprehensive measure.

This study's purpose was to develop and validate the Caregiver Evaluation of Quality of End of Life Care (CEQUEL) scale, a novel measure of perceived quality of EOL care that incorporates key Toolkit components with new measures of perceived suffering and prolongation of death. Caregiver data collected in the Coping with Cancer (CwC1) study were used to select relevant items for assessing the quality of EOL care, which were then analyzed to isolate core CEQUEL components and to determine CEQUEL's reliability and validity.

## Patients and Methods

### Ethics statement

Prior to the research being conducted, approval was obtained from the human subjects committees of all seven participating centers: Dana-Farber Cancer Institute (Boston, MA); Massachusetts General Hospital (Boston, MA); New Hampshire Oncology Hematology (Hookset, NH); Parkland Hospital (Dallas, TX); Simmons Comprehensive Cancer Center (Dallas, TX); Veterans' Affairs Connecticut Comprehensive Cancer Clinics (West Haven, CT); and Yale University Cancer Center (New Haven, CT). All participants provided written informed consent. Dr. Prigerson had full access to all of the data in the study and takes responsibility for the integrity of the data and the accuracy of the data analysis.

### Study design and sample

Coping with Cancer (CwC1) was an NCI- and NIMH-funded prospective, longitudinal, multi-site study of terminally ill cancer patients and their informal caregivers (e.g., spouse or adult child) followed through bereavement. Patients were recruited from September 1, 2002 to February 28, 2008 from seven outpatient sites in Texas, New York and New England. Approval was obtained from the human subjects committees of all participating centers; all enrolled patients provided written informed consent and received $25.

CwC1 patient eligibility criteria included an advanced metastatic cancer diagnosis, disease progression through chemotherapy, age ≥20 years, presence of an informal caregiver, absence of significant cognitive impairment, and English or Spanish proficiency. Eligible caregivers provided the majority of patients' unpaid, informal care. Research staff identified participants from weekly clinic rosters. Patients and caregivers were interviewed separately at baseline (Wave 1), and caregivers were interviewed again following patients' deaths (Wave 2). Additional information was obtained via chart review and post-mortem interviews with designated primary caregivers (N = 148; 57%) or with healthcare providers or others caring for patients at the time of death (N = 114; 43%).

The present report focuses on 275 patient/caregiver dyads with complete data for thirteen items used in the final model (initial sample = 315). Forty dyads with missing CEQUEL data did not differ significantly from those with full data on all examined demographic characteristics other than relationship to the patient (those identifying as “friend” were more likely to have missing information).

The median time from Wave 1 interview to death for the final analyzed cohort was three months, and from death to Wave 2 interview was 6.5 months.

### Scale development

The authors reviewed over 400 CwC1 Wave 2 items, identifying 69 related to caregiver perception of quality of EOL care. Item identification was based on relevant literature [8]–[15] as well as the authors' clinical judgment and research experience in psycho-oncology, EOL care, bereavement, and psychometrics. The item pool was further reduced to 21 by discarding redundant items, those related to patient care beyond the final week of life (so that all had the same time reference) and those inquiring about specific symptoms (for greater generalizability). Ten items were yes/no questions, and eleven were Likert scale questions. In order to achieve more balanced item distributions we dichotomized 4- and 10-point Likert items at midpoint and 7-point items using 4 as the split point (i.e. 1–4 = 1, 5–7 = 2). We reversed and/or recoded items as necessary to facilitate meaningful item summation, with “1” signifying perceived poorer quality of care and “2” perceived better quality of care.

### Caregiver demographics

Caregivers answered questions at Wave 1 about their own gender, age, race/ethnicity, marital status, income, education, religion and relationship to the patient.

### Items included in the factor analysis

The initial 21 items retained for factor analysis were all assessed in the Wave 2 bereaved caregiver interview. Twelve were adapted from the Toolkit, one from the Needs Near the End of Life Screening Tool (NEST) [37], and eight originated with CwC1.

### Correlates and outcomes

Select Wave 1, post-mortem and Wave 2 items were retained for convergent validity analysis [38], based on the hypothesis that all items would be significantly associated with CEQUEL scores.

Wave 1 patient items included patient baseline reports of advance care planning and EOL discussions with their physicians. In previous CwC1 studies, patient-provider discussion of EOL wishes was associated with less aggressive medical care, which was then associated with improved QOL in bereaved caregivers. Also retained were Wave 1 caregiver responses on the 14-item Brief RCOPE, a validated measure of positive and negative religious coping [39]. Negative religious coping has been associated with increased caregiver burden, poor mental health, and decreased QOL and satisfaction [40], [41]. Finally, patients answered Wave 1 questions about the degree to which they trusted and respected their physician, felt respected and “seen as a whole person” by their physician, and felt comfortable asking their physician questions about their care. Responses to these items were summed as a measure of “therapeutic alliance”, which has been previously identified as important to the QOL of dying patients and their families [1], [12].

Post-mortem items inquired about place of death, hospice enrollment, intensive care unit (ICU) admission and resuscitation. Prior research suggests that less aggressive medical care, dying on home hospice rather than in an ICU, and longer hospice enrollment are associated with better caregiver satisfaction with care, QOL and mental health [1], [10], [16]–[18], [42], [43].

Wave 2 caregiver items included questions related to caregiver regret, which has been inversely associated with perception of peaceful death [44]. Additional Wave 2 items were included to capture psychosocial distress in bereavement as an expected outcome of poor EOL care [33]. These include items from the Yale Evaluation of Suicidality (YES) scale [45], [46], the Stressful Caregiving Adult Response to Experience of Dying (SCARED) scale [47], the Beck Hopelessness Scale (BHS) [48], and the Structured Clinical Interview for DSM-IV (SCID) Axis I modules [49], [50].

### Statistical analyses

Descriptive statistics, means-difference testing, and correlational analyses were performed using SPSS Statistics, Version 19.0 (SPSS, Inc., 1989–2010). Analysis of the 21-item correlation matrix was conducted via exploratory factor analysis (EFA) techniques, using Mplus, Version 6.12 (Muthén & Muthén, 1998–2011). As suggested by Muthén et al. [51] for factor analysis with binary outcomes, a weighted least squares extraction method using tetrachoric correlations was employed. Item and factor retention was based on Muthén [52] criteria including Eigenvalues >1 [53], scree plot analysis [54], no negative residual variances, factor loading patterns, and substantive and theoretical interpretability. Parallel analysis [55] confirmed the appropriate number of factors. Model fit statistics were interpreted following Yu's [56] recommendations.

Items with factor loadings <0.4 were removed in successive factor analyses. Consecutive analyses were conducted until a 4-factor solution with clear factor loadings and good model fit was achieved. Final factor analysis items were summed and internal consistency was evaluated using Cronbach's α [57]. The non-normal distribution of CEQUEL scores required the use of nonparametric tests to evaluate demographic differences in CEQUEL scores as well as associations between CEQUEL scores and related EOL indicators.

## Results

### Sample

Table 1 provides relevant characteristics for the 275 caregivers used in this report. 76% were female, 70% were white, 68% were married, and 39% were Catholic. Caregivers ranged in age from 20 to 83 years (Mean = 51.9, Median = 53). 53% were the spouse or partner of the patient. 58% of caregivers in the present study were recruited from community-based sites. Mean CEQUEL scores were significantly lower (indicating poorer perceived quality of care) for Catholic than for non-Catholic caregivers (23.2 vs. 23.9, p = 0.015), as well as for caregivers reporting no religious affiliation compared to those with a religious affiliation (22.1 vs. 23.8, p = 0.021). Pentecostalists scored highest (Mean = 24.5), followed by Baptists (Mean = 24.3). CEQUEL scores did not vary significantly by other caregiver characteristics, but they did vary by recruitment site, with mean CEQUEL scores significantly lower for Yale caregivers than for those at both Simmons (22.8 vs. 24.5, p = 0.003) and Parkland (22.8 vs. 24.1, p = 0.001). This site difference remained significant at p<0.05 after controlling for religion as well as race.

**Table 1:** Demographic Characteristics of Sample Caregivers (N = 275).

Characteristic	No. caregivers		%
Sex^a^			
Male	64		24
Female	201		76
Age, years^a^			
Mean		51.9	
SD		13.6	
Race/ethnicity^b^			
White	185		70
Black	37		14
Asian-American, Pacific Islander, Indian	5		2
Hispanic	33		12.5
Other	4		1.5
Marital status^c^			
Married	172		68
Income^d^			
<$31,000	62		25
≥$31,000	123		50
Don't know	45		18
Declined	14		6
Education, years^b^			
Mean		13.5	
SD		3.6	
Religion^b^			
Catholic	102		39
Protestant	47		18
Baptist	36		14
Pentecostal	11		4
Jewish	13		5
Other	37		14
None	18		7
Relationship to patient^e^			
Spouse/partner	120		53
Son/daughter	57		25
Sibling	15		7
Other relative	17		7
Friend	6		2
Parent	11		5
Other	2		1
Recruitment site^f^			
Yale University Cancer Center	65		24
Veterans' Affairs Connecticut Comprehensive Cancer Clinics	13		5
Memorial Sloan-Kettering Cancer Center	18		6.5
Simmons Comprehensive Cancer Center	21		7.5
Parkland Hospital	89		33
Dana-Farber Cancer Institute	8		3
Massachusetts General Hospital	1		0.5
New Hampshire Oncology Hematology	56		20.5

Missing data: a: N = 265, b: N = 264, c: N = 253, d: N = 244, e: N = 228, f: N = 271.

### Factor analysis

**Figure 1:**
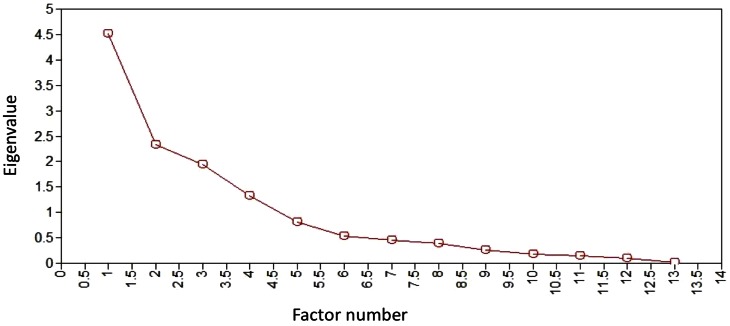
Scree plot of final four-factor, thirteen-item model.

Eigenvalue, scree-plot and parallel analyses all favored a 4-factor structure. Eight items with factor loadings <0.4 or with negative residual variances were dropped from successive models. Importantly, four of these were Toolkit items related to individual-focused care (e.g. patient being treated with respect and kindness). One item (“Was there any medical procedure or treatment that happened to patient that was inconsistent with his/her previously stated wishes?”) with a 0.39 factor loading was retained because its removal created model instability and because retention made substantive sense. Figure 1 shows the scree plot suggesting four factors for the final model, each with an Eigenvalue greater than 1.

Twelve of thirteen items loaded significantly on one of four identified factors (Table 2): Prolongation of Death (“Prolongation”), Perceived Suffering (“Suffering”), “Shared Decision-Making” and Preparation for the Death (“Preparation”). Toolkit items all loaded on Shared Decision-Making or Preparation, whereas CwC1-specific items all loaded on Prolongation or Suffering. Small, positive, significant correlations between most factors indicated that they represent four distinct aspects of a single construct. Fit statistics indicated good model fit.

**Table 2:** Factor Loading Scores and Fit Statistics for Final EFA Model.

	PROLONGATION OF DEATH	PERCEIVED SUFFERING	SHARED DECISION-MAKING	PREPARATION FOR THE DEATH	
1. Was the life of [PATIENT] prolonged by medical interventions longer than you would have wished?	0.848*				
2. Was the life of [PATIENT] prolonged by medical interventions when ___________ was, to the best of your knowledge, dying?	0.990*				
3. Was the life of [PATIENT] prolonged by medical interventions that resulted in an increase of his/her suffering?	0.843*				
4. How peaceful or violent did _____'s death seem to you?		0.708*			
5. To what extent do you think _________ suffered in dying?		0.953*			
6. How much did __________ suffer compared to what you expected?		0.846*			
7. Was there ever a problem understanding what any doctor was saying to you about what to expect from treatment?‡			0.698*		
8. Did you feel that the doctors you talked to listened to your concerns about [PATIENT'S] medical treatment?‡			0.881*		
9. Was there any medical procedure or treatment that happened to (him/her) that was inconsistent with (his/her) previously stated wishes?‡			0.390		
10. To the best of your knowledge, did [PATIENT'S] doctor or the medical staff who cared for (him/her) speak to (him/her) or you about (his/her) wishes about medical treatment?‡			0.548*		
11. How often were you or other family members kept informed about [PATIENT'S] condition?‡			(0.486)	0.562*	
12. Did you or your family receive any information about what to expect while (he/she) was dying?‡				0.668*	
13. At any time did you or your family receive any information about the medicines that would be used to manage (his/her) pain, shortness of breath, or other symptoms?‡				0.799*	
	**CFI**	**TLI**	**RMSEA**	**RMSR**	**X^2^**
EFA fit statistics	1.000	1.016	0.000	0.042	26.227 (p = 0.75)

* p≤.05.

‡ Toolkit After-Death Bereaved Family Member Interview.

### Psychometric properties of the CEQUEL scale

CEQUEL scores ranged from 16 to 26 out of a possible 26 points (M = 23.6, SD = 2.2, Median = 24), with higher scores signifying better perceived quality of care. One item – “How much did patient suffer compared to what you expected?” – had a slight majority reporting poorer perceived quality of care.

### Reliability

CEQUEL demonstrated an acceptable Cronbach's α of 0.69. Prolongation and Suffering had acceptable α's of 0.78 and 0.73, while Shared Decision-Making and Preparation had moderate α's of 0.52 and 0.54.

### Convergent validity

Table 3 illustrates patterns of association between CEQUEL and subscale scores and related EOL outcomes. In interpreting these associations, it is important to recall that higher CEQUEL and subscale scores reflect better perceived quality of care. Higher Prolongation and Suffering scores actually reflect *lower levels* of perceived prolongation and suffering (hence better quality of care within these domains).

**Table 3:** Patterns of Association between CEQUEL or CEQUEL subscales and key end-of-life outcomes (N = 275).

	CEQUEL			PROLONGATION			SUFFERING			SHARED DECISION-MAKING			PREPARATION		
	ρ	?^2^	z	ρ	?^2^	Z	ρ	?^2^	z	ρ	?^2^	z	ρ	?^2^	z
***Wave 1 patient items***															
Do you have a signed Living Will/Health Care Proxy/Durable Power of Attorney for health care/all or none?^1^			−.095			−.609			−.746			−.448			−.256
Have you completed a Do Not Resuscitate (DNR) order?^2^			−1.82			−1.17			**−1.96** ***			−1.54			−.57
Have you and your doctor discussed any particular wishes you have about the care you would want to receive if you were dying?^1^			−.103			−.92			−.92			−.56			−.05
Therapeutic alliance	**.129** ***			.094			.114			.089			.060		
***Wave 1 caregiver items***															
Positive RCope^3^	.105			−.017			.016			.113			**.149** ***		
Negative Rcope^3^	−.117			**−.126** ***			**−.155** ***			.037			.037		
***Post-mortem items***															
Where did the patient's death take place?^4^		9.67			6.76			6.09			10.41			9.33	
Was inpatient hospice involved in the care of (PATIENT), so that (he/she) stayed in a hospice facility?^5^			−1.27			−1.44			−1.19			−.09			−.46
For about how long did (PATIENT) get inpatient hospice care before (his/her) death?^6^		**11.80** **			5.53			**10.45** **			3.72			2.84	
Was outpatient hospice involved in the care of (PATIENT), so that a hospice worker cared for (him/her) in the home?^7^			**−2.09** ***			−1.52			−.05			**−2.34** ***			**−3.02** **
For about how long did (PATIENT) get outpatient hospice care before (his/her) death?^8^		4.61			4.64			4.95			3.34			.46	
Was the patient in the Intensive Care Unit in the week leading up to his/her death?^7^			−1.18			−1.01			−.22			−1.36			−1.30
Was the patient resuscitated in the week leading up to the death?^7^			−.54			−.58			−.13			−.31			−.30
***Wave 2 bereaved caregiver items***															
On a scale of 1 to 5, how would you rate your regrets about how (PATIENT) died?^9^	**−.359** *			**−.211** *			**−.340** *			**−.144** ***			−.042		
On a scale of 1 to 5, how would you rate your regrets about the care provided by clinicians to (PATIENT) just prior to his/her death?^10^	**−.434** *			**−.178** **			**−.357** *			**−.321** *			**−.216** *		
On a scale of 1 to 5, how would you rate your regrets about the care you were able to provide to (PATIENT) just prior to his/her death?^11^	**−.214** *			**−.148** ***			**−.154** **			**−.158** **			**−.135** ***		
In light of current circumstances, how strong would you say your wish to die has been?^10^	−.029			−.025			−.033			.002			.069		
In light of your current circumstances, have you ever had thoughts of killing yourself?^12^	−.113			−.082			−.070			−.024			−.040		
Felt __________ had had enough^13^	**−.176** **			**−.182** **			−.076			−.089			.016		
How fearful related to this?^14^	**−.185** **			**−.134** ***			**−.210** *			−.095			.094		
How helpless related to this?^3^	−.096			−.069			−.124			−.079			.110		
Beck Hopelessness Scale^15^	−.085			−.183			−.074			−.032			.118		
Bereavement Challenges Scale^16^	−.032			−.034			.031			−.071			.003		
MDD^4^			−1.30			−1.10			−.10			**−2.49** **			−.38
PTSD^7^			**−2.06** ***			**−3.90** *			−.36			−.71			−.12
GAD^17^			−.14			−.72			−.85			−.28			−1.52
PD^4^			−1.30			−.02			−.57			−1.81			−.83

Asymptotic significance levels:

* p≤.001,

** p≤.01,

*** p≤.05.

Statistical tests: ρ: Spearman's rho correlation coefficient, χ^2^: Kruskall-Wallis test, z: Mann-Whitney U test.

Missing data: 1: N = 250, 2: N = 248, 3: N = 237, 4: N = 261, 5: N = 259, 6: N = 49, 7: N = 260, 8: N = 176, 9: N = 274, 10: N = 273, 11: N = 272, 12: N = 270, 13: N = 256, 14: N = 238, 15: N = 93, 16: N = 179, 17: N = 257.

#### Wave 1 patient items

Higher Suffering scores (indicating less perceived suffering) were positively associated with baseline completion of a DNR order (p≤.05). There were no other significant differences in CEQUEL or subscale scores based on baseline advance care planning. Higher CEQUEL scores were significantly positively associated with therapeutic alliance (p≤.05).

#### Wave 1 caregiver items

Higher Preparation scores were significantly associated with higher levels of positive religious coping (p≤.05) and higher Prolongation and Suffering scores (i.e. less perceived prolongation and suffering) were associated with lower levels of negative religious coping (p≤.05).

#### Post-mortem items

There were no significant differences in CEQUEL or subscale scores based on location of death, ICU admission, resuscitation or receipt of inpatient hospice care. Higher CEQUEL and Suffering scores were positively associated (p≤.01) with length of inpatient hospice enrollment. Higher CEQUEL (p≤.05), Shared Decision-Making (p≤.05) and Preparation (p≤.01) scores were positively associated with receipt of home hospice care, but not length of enrollment.

#### Wave 2 bereaved caregiver items

Higher CEQUEL and subscale scores were negatively associated with regret. Higher CEQUEL and Prolongation scores were negatively associated with feeling that the patient had had enough (p≤.01) and related fear. Finally, higher Shared Decision-Making scores were negatively associated with meeting criteria for Major Depressive Disorder (p≤.01), and higher CEQUEL (p≤.05) and Prolongation (p≤.001) scores were negatively associated with meeting criteria for Posttraumatic Stress Disorder.

## Discussion

The present analysis suggests that CEQUEL is a valid measure of quality of care at the EOL from the perspective of cancer patient caregivers. CEQUEL's thirteen items comprise four distinct but related factors that are consistent with both the literature and clinical practice related to EOL care: Prolongation of Death, Perceived Suffering, Shared Decision-Making, and Preparation for the Death. Perceived or actual inadequacy within any of these domains is associated with heightened caregiver risk for poor bereavement outcomes, but existing measurement tools do not capture caregiver perceptions of suffering and prolongation of death. Their inclusion in CEQUEL, together with the scale's relative brevity, extends its clinical relevance and utility beyond existing instruments.

This study suggests that CEQUEL has strong convergent validity. Higher CEQUEL scores were positively associated with home hospice enrollment, as well as length of inpatient hospice enrollment. Higher CEQUEL scores were negatively associated with bereaved caregiver regret and with psychological trauma symptoms. Convergence with these post-loss indicators suggests that CEQUEL measures aspects of the caregiver experience that are of critical import not only during the dying process but also in post-loss adjustment. Finally, positive associations between CEQUEL scores and patient-physician therapeutic alliance are consistent with previous research demonstrating that therapeutic alliance results in less aggressive, burdensome EOL care and improved patient mental health [1], [58]. Taken together, these findings are consistent with the broader literature and support CEQUEL's validity as a measure of perceived quality of EOL care.

A unique contribution of CEQUEL is its inclusion of suffering and prolongation of death as key indicators of caregiver-perceived quality of care, and this report found data suggesting the importance of both. Lower Prolongation and Suffering scores (i.e. higher levels of perceived prolongation and suffering), were positively associated with caregiver regret, fear and negative religious coping. This finding is significant in light of the association between negative religious coping and caregiver mental health and QOL outcomes [39]. Higher Prolongation scores were also negatively associated with trauma symptoms, whereas lower Suffering scores were negatively associated with DNR orders and length of inpatient hospice enrollment. These unique associations highlight the importance of assessing for caregiver perceptions of suffering and prolongation of death in terminal care. The fact that caregivers identified the poorest perceived quality of care within the three Suffering items further speaks to this domain's influence on caregiver wellbeing.

The present study suggests several directions for further study. CEQUEL's reliability and validity need to be confirmed in non-cancer patient and caregiver samples, as this population may interpret quality of care at the EOL differently. While CwC1 recruitment sites included a VA hospital and two community-based sites (Parkland and NHOH) that accounted for 58% of the total present sample, the study also included several academic medical centers that might be more inclined towards or capable of aggressive interventions, including trial participation. Interestingly, there was no clear relationship between care setting (community-based vs. academic) and CEQUEL scores in the present sample, with mean CEQUEL scores as follows: Simmons, 24.5; Parkland, 24.1; DFCI, 24.1; MSKCC, 23.9; CT VA, 23.4; NHOH, 23.4; and Yale, 22.8. Hospice enrollment at time of death was higher among CwC1 participants (63%) than for total US deaths (45%) in 2011 [59], but the proportion of hospice patients dying at home was quite similar between CwC1 (70%) and the US (66%), as were deaths on inpatient hospice units (CwC1: 19%, US: 26%). Taken together, these data suggest CwC1 data provided a fairly representative sampling of patients. Nevertheless, these results should be confirmed with more recent data.

Future iterations of CEQUEL might also include more straightforward language for some items, such as item 5 (see Appendix S1). There was one item that used relatively simpler language to address the same concern of adherence to patient wishes (“During the last week of life, to what extent were patient's wishes followed regarding a course of treatment that focused on extending life as much as possible even if it meant more pain and discomfort, or on a plan of care that focused on relieving pain and discomfort as much as possible even if it meant not living as long?”) but this item could not be retained due to negative error variance. Further refinement of CEQUEL should strive for scale items that are psychometrically sound but also simply stated. Researchers have questioned the reliability of retrospective data collected via post-death interviews rather than during the dying process [6], [7]. However, assessing caregiver perceptions of EOL care in ‘real time’ is not only impractical (i.e. knowing when patients are dying and being able to make concurrent assessments), but also ethically questionable (e.g., it pulls vulnerable caregivers away from the bedside when they may feel that their exclusive focus should be on the patient). Furthermore, bereavement experts are familiar with the tendency of caregivers to recall their loved ones' final days in excruciating detail for months to years into bereavement. Future research will need to compare the reliability of caregiver reports taken in the first few months of bereavement compared to six months post-loss.

Finally, our finding that Catholic caregivers and those with no religious affiliation scored worse than other groups on CEQUEL merits further examination of potential reasons for this disparity. One clue may lie in the use of religious coping. Predictably, caregivers with no religious affiliation were significantly less likely to use positive religious coping than any other group. Catholic caregivers, however, also used significantly less positive religious coping than Baptists, Pentecostalists or those selecting “Other” as their faith affiliation (including Muslim but excluding Protestant or Jewish). Perhaps this relative lack of a positive and loving connection with a higher power acts as a detriment to positive coping in general. The way in which Catholics perceive or cope with care in the final week of life may also be affected by internal conflict with the Church's teachings on redemptive suffering, or the Church's tension around withholding or withdrawing life-sustaining treatments. Caregivers with no religious affiliation may be at a disadvantage relative to those who can rely on an extra layer of support via their religious community, or a religious framework that lends broader meaning to times of crisis and loss. Future studies should move beyond hypotheticals and strive for concrete data to help explain why religious faith, or lack thereof, influences caregiver's perceptions of the quality of care provided to their dying loved one.

This study's findings are notable from a research perspective, but their clinical implications for social workers and other healthcare providers are equally important. A low pre-loss CEQUEL score may prompt a caregiver-team meeting in which caregiver expectations about preventing a prolonged death or mitigating perceived suffering are weighed against what is achievable, and redirection of care or reframing of caregiver interpretations are pursued as necessary. Clinicians in day-to-day practice are likely to overlook some of the key questions addressed in CEQUEL, resulting in situations where caregivers either ‘act out’ or suffer silently without the team understanding why. CEQUEL helps to identify these underlying causes of distress and, to the extent that these issues are effectively addressed, may mitigate caregiver-team conflict or poor bereavement outcomes. Similarly, post-loss CEQUEL administration may facilitate bereavement adjustment by enabling clinicians to identify, reframe and process underlying sources of regret, trauma or other distress. Each CEQUEL factor represents a component of care that may leave caregivers feeling like the team should have done something differently, or that caregivers themselves have failed their loved ones. Associations between CEQUEL scores and caregiver regret, including regrets about their own role in the final week of life, highlight this potential. The literature on caregiver regret in bereavement is limited, but suggests that regret resolution leads to improved bereavement outcomes [60]. Minimizing caregiver regret is one way to reduce suffering in bereaved caregivers, and CEQUEL provides clinicians with one way to identify caregivers at risk for post-loss regret and other bereavement sequelae. Our findings that low CEQUEL scores, as well as perceived prolongation of death and suffering subscales, are associated with negative bereavement outcomes will likely be of general interest to those caring for the dying, but perhaps particularly to advocates for physician aide in dying (PAD). CwC1 did not address PAD and it is not the intent of the present study to argue for or against it. Our findings may have implications for this debate, however, particularly in light of recent findings that patients pursuing PAD often do so out of concern for lost autonomy, dignity and functional status, and that bereaved family members of these patients feel more certain that their loved ones' wishes were honored, more prepared for and accepting of the death, and less regretful about the circumstances of death [61], [62].

The results of this study suggest that CEQUEL is a reliable and valid tool for assessing caregiver perceptions of the quality of EOL care provided to dying cancer patients. By including novel dimensions of suffering and prolongation of death, we have developed an assessment tool that more fully captures perceived deficiencies in EOL care. CEQUEL appears to identify important targets for clinical intervention that can improve EOL outcomes not only during terminal care but also in caregivers' subsequent bereavement.

## Supporting Information

Appendix S1 **Caregiver Evaluation of Quality of End-of-Life Care (CEQUEL).**
(DOCX)
