# Correction: Caregiver Evaluation of the Quality of End-Of-Life Care (CEQUEL) Scale: The Caregiver's Perception of Patient Care Near Death

**DOI:** 10.1371/annotation/c427c4ef-bc6f-41d8-9951-7efcff550171

**Published:** 2013-09-19

**Authors:** Philip C. Higgins, Holly G. Prigerson

An affiliation of the first author was incorrectly omitted. Philip C. Higgins is not only affiliated with institutions numbers 1-3, but also Department of Care Coordination, Brigham & Women's Hospital and the Dana-Farber/Brigham & Women's Cancer Center. 

