# Supplementary material for: Caregiver Evaluation of the Quality of End-Of-Life Care (CEQUEL) Scale: The Caregiver's Perception of Patient Care Near Death
Source: PLoS One. 2013 Jun 6;8(6):e66066. doi: 10.1371/journal.pone.0066066 (PMC3675191; doi:10.1371/journal.pone.0066066)
Supplement: Appendix S1 — Caregiver Evaluation of Quality of End-of-Life Care (CEQUEL). (DOCX) [file pone.0066066.s001.docx]

| **Caregiver Evaluation of Quality of End-of-Life Care (CEQUEL)** | | |
| --- | --- | --- |
|  | **YES (1 POINT)** | **NO (2 POINTS)** |
| **1. Was the life of [PATIENT] prolonged by medical interventions longer than you would have wished?** |  |  |
|  | **YES (1 POINT)** | **NO (2 POINTS)** |
| **2. Was the life of [PATIENT] prolonged by medical interventions when ___________ was, to the best of your knowledge, dying?** |  |  |
|  | **YES (1 POINT)** | **NO (2 POINTS)** |
| **3. Was the life of [PATIENT] prolonged by medical interventions that resulted in an increase of his/her suffering?** |  |  |
|  | **YES (1 POINT)** | **NO (2 POINTS)** |
| **4. Was there ever a problem understanding what any doctor was saying to you about what to expect from treatment?** |  |  |
|  | **YES (1 POINT)** | **NO (2 POINTS)** |
| **5. Was there any medical procedure or treatment that happened to (him/her) that was inconsistent with (his/her) previously stated wishes?** |  |  |
|  | **NO (1 PT)** | **YES (2 PT)** |
| **6. Did you feel that the doctors you talked to listened to your concerns about [PATIENT’S] medical treatment?** |  |  |
|  | **NO (1 PT)** | **YES (2 PT)** |
| **7. To the best of your knowledge, did [PATIENT’S] doctor or the medical staff who cared for (him/her) speak to (him/her) or you about (his/her) wishes about medical treatment?** |  |  |
|  | **NO (1 PT)** | **YES (2 PT)** |
| **8. Did you or your family receive any information about what to expect while (he/she) was dying?** |  |  |
|  | **NO (1 PT)** | **YES (2 PT)** |
| **9. At any time did you or your family receive any information about the medicines that would be used to manage (his/her) pain, shortness of breath, or other symptoms?** |  |  |
|  | **SOMETIMES/NEVER (1 PT)** | **USUALLY/ALWAYS (2 PT)** |
| **10. How often were you or other family members kept informed about [PATIENT’S] condition?** |  |  |
|  | **EXTREMELY (1 PT)** | **MINIMALLY (2 PT)** |
| **11. To what extent do you think _________ suffered in dying?** |  |  |
|  | **VIOLENT (1 PT)** | **PEACEFUL (2 PT)** |
| **12. How peaceful or violent did _____’s death seem to you?** |  |  |
|  | **MUCH MORE (1 PT)** | **MUCH LESS (2 PT)** |
| **13. How much did __________ suffer compared to what you expected?** |  |  |
